# Contemplating the Need to Advance Cardiac Device Algorithms

**DOI:** 10.19102/icrm.2019.101003

**Published:** 2019-10-15

**Keywords:** Capture management, cardiac implantable electronic device, cardiac resynchronization therapy

## Drs. Foo and Stiles discuss

Automatic threshold management algorithms in modern cardiac implantable electronic devices (CIEDs) have helped to extend device battery life.^1–5^ In general, these algorithms have been tested in standard endocardial systems; however, their use and safety in epicardial systems have not been as well-investigated. The presented case by Johnsrude et al.^6^ highlights the potential pitfalls of automated algorithms in systems with known or potential lead issues.

The case describes periods of asystole due to the temporary substitution of right ventricular (RV)-only pacing for biventricular pacing during automatic atrial threshold testing in a patient with a cardiac resynchronization therapy pacemaker (CRT-P; Medtronic, Minneapolis, MN, USA) with epicardial RV lead failure. Automatic atrial threshold algorithms are available in CIEDs of all of the major device companies with the exception of those from Microport CRM (Clamart, France). Notably, the possible inclusion of RV-only pacing in place of biventricular pacing during automatic atrial threshold testing is not limited to Medtronic devices. Rather, this peculiarity applies to most devices with automatic atrial threshold algorithms apart from devices from Abbott Laboratories (Chicago, IL, USA) and some selected Biotronik (Berlin, Germany) and Medtronic models. Automatic atrial threshold algorithms are nominally “on” at the time of implant in devices from the latter two companies with the capability. Although devices from Boston Scientific (Natick, MA, USA) and Abbott Laboratories are nominally set to “monitor” only at implant, atrial threshold testing is still performed (and, thereby, biventricular pacing is suspended in Boston Scientific devices) without adjustment to atrial pacing outputs **(Table 1)**.

In the clinical scenario described by the authors, even if was possible to program left ventricular (LV)-only pacing or to maintain biventricular pacing during atrial threshold testing, a functioning RV lead is still required in the vast majority of devices, as LV pacing timing is still dependent on RV sensed events. Hence, lead noise from a failing RV lead could lead to unpredictable LV pacing during atrial threshold testing.

As the authors have discovered, these issues are not widely recognized. In preparing this editorial, our informal survey of device company representatives, cardiac physiologists, and implanting physicians at high-volume cardiac device implant centers in New Zealand found variable awareness existed regarding these issues. When connecting a pacemaker generator to epicardial leads, the decision to alter the nominal programming of these algorithms varies across implant centers in New Zealand. Some implant centers are guided by advice from device company representatives or evaluate each implant on a case-by-case basis, while others choose to turn off all automated threshold algorithms in epicardial systems at implant due to concerns regarding early lead failure. However, once an RV lead has been identified to be failing, it is considered a best practice to turn off all automatic threshold algorithms due to concerns regarding the reliability of those algorithms in that setting. With the recent interest in His-bundle pacing and the complexity of such systems, the clinical impact of automatic atrial threshold algorithms must be carefully considered when used in combination with nonstandard device systems.

Epicardial leads have a higher rate of lead failure, which can be difficult to predict.^7–10^ At the time of the last surgery in the present case, the patient’s RV lead was a 10-year old unipolar epicardial lead. This case highlights the difficult decisions faced during device revision—does one use suboptimal existing leads and risk future lead failure or attempt the implantation of a new lead with all the attendant morbidity of the extra lead implant procedure? These questions are never easy and, sometimes, the answer is only known in retrospect. We commend the authors of this case in bringing this issue to the attention of the device community.

Fang Shawn Foo, md (shawnfoo@icloud.com)^1^ and Martin K. Stiles, mbchb, phd, fracp, fcsanz, fhrs^2^

^1^Department of Cardiology, North Shore Hospital, Auckland, New Zealand

^2^Department of Cardiology, Waikato Hospital, Hamilton, New Zealand

The authors report no conflicts of interest for the published content.

References1.ChenRChenKWangFHuaWZhangSImpact of automatic threshold capture on pulse generator longevity.Chin Med J (Engl).200611911925929[PubMed]167807722.GelvanDCrystalEDokumaciBGoldshmidYOvsyshcherIEEffect of modern pacing algorithms on generator longevity: a predictive analysis.Pacing Clin Electrophysiol.200326917961802[CrossRef][PubMed]1293049210.1046/j.1460-9592.2003.t01-1-00272.x3.SperzelJMilasinovicGSmithTWAutomatic measurement of atrial pacing thresholds in dual-chamber pacemakers: Clinical experience with atrial capture management.Heart Rhythm.200521112031210[CrossRef][PubMed]1625391010.1016/j.hrthm.2005.07.0234.CohenMBuckKTanelRECapture management efficacy in children and young adults with endocardial and unipolar epicardial systems.Europace.200463248255[CrossRef][PubMed]1512107910.1016/j.eupc.2004.02.0015.HiippalaASerwerGAClaussonEDavenportLBrandTHapponenJMAutomatic atrial threshold measurement and adjustment in pediatric patients.Pacing Clin Electrophysiol.2010333309313[CrossRef][PubMed]1995450510.1111/j.1540-8159.2009.02619.x6.JohnsrudeCLLauKCA cautionary tale on atrial capture management, biventricular pacing, and recurrent asystole.J Innov Cardiac Rhythm Manage.201910103848385210.19102/icrm.2019.101001PMC7252737324777047.CohenMIBushDMVetterVLPermanent epicardial pacing in pediatric patients: seventeen years of experience and 1200 outpatient visits.Circulation.20011032125852590[CrossRef][PubMed]1138272810.1161/01.cir.103.21.25858.MurayamaHMaedaMSakuraiHUsuiAUedaYPredictors affecting durability of epicardial pacemaker leads in pediatric patients.J Thorac Cardiovasc Surg.20081352361366[CrossRef][PubMed]1824226910.1016/j.jtcvs.2007.09.0029.PostMCBudtsWVan de BruaeneAFailure of epicardial pacing leads in congenital heart disease: not uncommon and difficult to predict.Neth Heart J.2011197–8331335[CrossRef][PubMed]2156721710.1007/s12471-011-0158-5PMC314432710.FortescueEBBerulCICecchinFWalshEPTriedmanJKAlexanderMEPatient, procedural, and hardware factors associated with pacemaker lead failures in pediatrics and congenital heart disease.Heart Rhythm.200412150159[CrossRef][PubMed]1585114610.1016/j.hrthm.2004.02.020

## Dr. Knilans considers

One could argue that the quote “life is really simple, but we insist on making it complicated,” attributed to Confucius in 500 B.C., is applicable to device algorithms in modern CIEDs. The first implanted cardiac pacemakers were incapable of sensing and had no ability for interrogation or programming. At that point, the addition of functionality despite its associated complexity was beneficial for patients. Collaborations between engineers and physicians have led to the introduction of devices with incredible capabilities but required convolution. Physicians trained in cardiology are familiar with the North American Society of Pacing and Electrophysiology Mode Code Committee/British Pacing and Electrophysiology Group pacemaker code, although most have shunned the 2002 revision^1^ and still refer to the simpler 1987 code.^2^ Those trained in cardiac electrophysiology are even more familiar with the nuances of “ventricular safety pacing” and other, more obscure algorithms that have enhanced device functionality and safety. However, very few physicians understand the specifics of device algorithms such as the “capture management” algorithm described in the report by Johnsrude et al.^3^ As suggested in the case report, this lack of knowledge can have potentially devastating consequences.

In the last 30 years, there has been a proliferation of these algorithms in the industry. They have developed in a manner akin to an “arms race” by manufacturers trying to create “a better mouse-trap.” Unfortunately, the various algorithms that exist are for the most part proprietary and there is no agreed-upon “international standard” at this time. This adds additional complexity for practitioners attempting to decipher different formulas from different manufacturers or even on different platforms from the same manufacturer that are designed to deal with the same concern. On most devices, these algorithms are nominally programmed to an “on” status. From the inception of our device clinic, our practice has been to turn off all algorithms at the time of implant unless we feel that the patient will benefit from them. We can then turn them on as indicated by the evolution of the patient’s specific situation. As demonstrated in the aforementioned case report, even an algorithm as seemingly innocuous as “capture management” can have significant consequences. The level of complexity is further illustrated by the fact that the patient’s asystole was the result of atrial and not ventricular capture management.

As illustrated in the case, many of these monitoring algorithms are programmed to function in the early morning hours, presumably so as not to disturb the patient. In my experience, the effects are often discovered when patients are admitted to the hospital on “full-disclosure” monitoring or when ambulatory electrocardiography is performed. Unfortunately, the use of ambulatory electrocardiography in patients with CIEDs has become quite uncommon, as many clinicians have been lulled into a sense of security by the availability of internal device monitoring capabilities.

Deleterious effects of CIED algorithms could be mitigated using a few simple steps. First, unless there is a proven benefit of an algorithm in a large population outweighing the deleterious effects, manufacturers should nominally program all algorithms to “off.” Second, all algorithms should be able to be tested in a clinic setting, similar to the performance of a sensing or capture threshold test, while the patient is being monitored so that the clinician can observe the effects. Third, clinicians should turn off all algorithms that they do not think are offering specific benefits to the patient and strongly consider incorporating ambulatory monitoring after the initial implantation of a device or after activating an algorithm. As further changes in devices will likely be a long-time coming (if ever), this third step will be the most important now.

The life of the man described in this case report may well have been saved by the presence of a redundant ventricular lead. He was at risk for a fatal event during the two-year period between reprogramming of the RV lead output to minimal settings and the discovery of the capture management issue. He may remain at risk, as the replacement of a new redundant lead was not described in the report. After witnessing the sudden death of a pacemaker-dependent patient from lead fracture during my fellowship training, I have always considered including a redundant lead for “back-up” pacing in my patients who are pacemaker-dependent. Prior to the development of biventricular pacemakers, this meant the placement of two pacemakers, but, with the relatively high failure rate of pacing leads in pediatric patients, it didn’t take long to see patients in whom this approach was either life-saving or at least avoided a significant symptomatic event. Sudden death in device-dependent patients may be related to continued asystole or to ventricular fibrillation following a period of asystole.^4,5^ Presenting definitive proof that the inclusion of redundant pacing is life-saving is somewhat challenging, but a report published by Ceresnak et al.^6^ in 2015 suggested a reduction of cardiovascular events in pediatric patients with redundant leads when a lead failed.

Even though it will likely require an open-chest procedure, placement of a redundant ventricular lead, in my opinion, would be advisable for the man in the presented case report as well as for most pacemaker-dependent patients in the pediatric age range and those with epicardial leads due to the high lead failure rate in these groups. Further, it should really be considered in all pacemaker-dependent patients of any age and any lead type, and the approach should at least be mentioned in guidelines for bradycardia pacing (which has never before occurred).^7^

Timothy K. Knilans, md (timothy.knilans@cchmc.org)^1,2^

^1^Department of Pediatrics, Division of Cardiology, Cincinnati Children’s Hospital Medical Center, Cincinnati, OH, USA

^2^University of Cincinnati College of Medicine, Cincinnati, OH, USA

The author reports no conflicts of interest for the published content.

References1.BernsteinADDaubertJCFletcherRDThe Revised NASPE/BPEG generic code for antibradycardia, adaptive-rate and multisite pacing.Pacing Clin Electrophysiol.2002252260264[CrossRef][PubMed]1191600210.1046/j.1460-9592.2002.00260.x2.BernsteinADCammAJFletcherRDThe NASPE/BPEG generic pacemaker code for antibradyarrhythmia and adaptive-rate pacing and antitachyarrhythmia devices.Pacing Clin Electrophysiol.1987104 Pt 1794799[CrossRef][PubMed]244136310.1111/j.1540-8159.1987.tb06035.x3.JohnsrudeCLLauKCA cautionary tale on atrial capture management, biventricular pacing, and recurrent asystole.J Innov Cardiac Rhythm Manage.201910103848385210.19102/icrm.2019.101001PMC7252737324777044.UndaviaMFischerAMehtaDFatal outcome in a pacemaker-dependent patient.Pacing Clin Electrophysiol.200932455055310.1111/j.1540-8159.2009.02320.x193358695.YesilMBayataSPostaciNPacemaker inhibition and asystole in a pacemaker dependent patient.Pacing Clin Electrophysiol.199518101963196410.1111/j.1540-8159.1995.tb03846.x85391666.CeresnakSRPereraJLMotonagaKSVentricular lead redundancy to prevent cardiovascular events and sudden death from lead fracture in pacemaker-dependent children.Heart Rhythm.2015121111116[CrossRef][PubMed]2527798810.1016/j.hrthm.2014.09.0567.KusomotoFMSchoenfeldMHBarrettC2018 ACC/AHA/HRS guideline on the evaluation and management of patients with bradycardia and cardiac conduction delay.Heart Rhythm.2018 Nov 6pii: S1547-5271(18)31127-5. [CrossRef][PubMed]10.1016/j.hrthm.2018.10.03630412777

## Drs. Patel and Tanel evaluate

The case reported by Johnsrude et al.^1^ highlights the unique complexities of cardiac rhythm management in pediatric and congenital heart disease patients and the importance of understanding these unique events to improve overall device programming and safety for all patients. Here, potentially dangerous asystolic events due to a “perfect storm” of underlying conduction disease (complete heart block), device type (cardiac resynchronization therapy pacemaker; CRT-P), device functionality (failed/”turned off” RV lead), and device programming (Atrial Capture Management™; Medtronic, Minneapolis, MN, USA) resulted in failed ventricular capture. It was serendipitous that the patient was placed on inpatient telemetry for a neurology evaluation, as the malfunction attributed to the algorithm in question only manifested during sleep, which is when the device normally runs said algorithm. The identification of this issue raises a few clear practical considerations that the authors highlight regarding device programming, monitoring, and device setup.

The identification of device programming problems that may arise requires a thorough understanding of the “bells and whistles” that newer device models have. The outcome of this device programming issue might have been avoided with this knowledge. Alternatively, the outcome could be prevented using a relatively simple software solution. There are certainly many alerts during the course of implantable device programming that communicate potentially dangerous situations to the health care provider who is programming and monitoring the device. One potential solution is for an alert to occur when the Atrial Capture Management™ algorithm is enabled that would remind the clinician that RV lead-only pacing would occur while the capture threshold test is being performed. Alternatively, when the Atrial Capture Management™ algorithm is engaged, there could be a programming hard-stop to prevent the RV lead from being “turned off”/ programmed subthreshold. In hindsight, the use of the Atrial Capture Management™ algorithm in this patient with only 13% atrial pacing was unlikely to provide a substantial benefit in extending battery longevity, and turning on this optional feature resulted in unintended consequences. In addition, placing the more recently implanted and better-performing LV lead in the RV port of the CRT-P device could have averted the problem that was encountered. As is common with congenital heart disease patients, the port and lead functionality should dictate pairing rather than lead location and port label.

The assessment of device performance generally has relied on device interrogation rather than the use of ambulatory electrocardiographic monitoring unless prompted by clinical concerns or device interrogation abnormalities. At the time of implantation of cardiac rhythm devices in the setting of pediatric or congenital heart disease, patients are often observed using telemetry/cardiac rhythm monitoring postimplantation, which can provide short-term information regarding device functionality. However, features such as capture management are generally turned on automatically or in-person after the acute implant phase (after the first one to three months), which, as demonstrated in this patient, does not allow for the identification of the observed consequence with routine device follow-up. At our institution, we do not necessarily perform routine ambulatory cardiac rhythm monitoring in all cardiac rhythm management device patients. Additional monitoring is prompted by signs or symptoms concerning for bradycardia or pacemaker failure, signs or symptoms suggestive of arrhythmias not recorded by the device, a need for further rhythm/arrhythmia discrimination, or concerns about device malfunction/lead failure. The most difficult question remains not if but when and for how long monitoring should be performed. For malfunctions that occur every night, like in the case described by Johnsrude et al.,^1^ a simple 24-hour Holter monitor would be sufficient. Unfortunately, if the abnormality occurs less frequently, more detailed and longer-term monitoring would be necessary. There are no published data to help support practice guidelines regarding ambulatory cardiac monitoring after device implantation. This is likely a consequence of the many safety features/alerts that cardiac rhythm devices currently have. However, as devices become even more complex, there will always remain the potential for these “perfect storms” that result in rare pacemaker “malfunctions” that may only be identified by ambulatory cardiac monitoring. Therefore, it is critical to make every effort to understand existing device algorithms.

Device placement in patients with complex congenital heart disease can be limited and challenging due to the anatomy or multiple surgical procedures during childhood. The case described by Johnsrude et al.^1^ is not surprising. Patients with multiple re-do sternotomies have extensive epicardial scar that makes placing epicardial leads a challenge. Sites for implantation may be limited and capture thresholds may be high. Exit block is not unusual, and delayed conduction through diseased myocardium may make pacing from some sites suboptimal with regard to timing.^2–4^ In young children, the size of the heart often results in far-field oversensing of adjacent chambers. Finally, young patients are generally more active and sustain injuries or perform repetitive motions that can result in lead insulation breakage or lead fracture. For all of these reasons, pacing systems may be less durable and result in shorter battery longevity than is desired in these young individuals who are anticipated to have many years of pacemaker dependence ahead of them. Therefore, health care providers must often craft innovative and alternative, “off-label” uses of hardware. Here, the authors used a CRT-P device to provide a redundant ventricular lead in the setting of pacemaker dependence for complete atrioventricular block with an unreliable escape. This concept of a back-up pacing option is also performed using two devices (one transvenous and one back-up epicardial), a dual-chamber device with a second ventricular lead placed in the atrial port, or a CRT-P device as discussed here. However, when using devices in nontraditional ways, we must recognize these limitations, pay vigilant attention, and disseminate recognized shortcomings and possible untoward effects from these practices.

Finally, transvenous systems are often regarded as superior to epicardial systems but may not be a realistic option for young patients or patients with complex congenital heart disease. More importantly, these cohorts of patients are not immune to similar problems, as noted by the authors. Although a head-to-head comparison of the longevity and survival of epicardial and endocardial leads in pediatric and congenital heart disease patients has not been performed in some time, the conventional understanding that epicardial leads fail at a higher rate is not necessarily always true.^5,6^ Beaufort-Krol et al. found that steroid-eluting epicardial leads had the same longevity as conventional endocardial leads. Further, the pacing and sensing thresholds were similar and did not change during follow-up. Therefore, steroid-eluting epicardial pacing leads were deemed to be a good alternative in small children and children with congenital heart disease.

Ultimately, the case report of Johnsrude et al. is a sobering lesson to remind us that some off-label practices in the most complex patients may have unintended deleterious effects. A careful understanding of the tools that we use must be taken into consideration, especially when some of the possible risks have not been previously experienced or reported.

Akash R. Patel, md^1,2^ and Ronn E. Tanel, md (ronn.tanel@ucsf.edu)^1,2^

^1^Department of Pediatrics, UCSF School of Medicine, San Francisco, CA, USA

^2^Pediatric and Congenital Arrhythmia Service, UCSF Benioff Children’s Hospital, San Francisco, CA, USA

The authors report no conflicts of interest for the published content.

References1.JohnsrudeCLLauKCA cautionary tale on atrial capture management, biventricular pacing, and recurrent asystole.J Innov Cardiac Rhythm Manage.201910103848385210.19102/icrm.2019.101001PMC7252737324777042.CohenMIBushDMVetterVLPermanent epicardial pacing in pediatric patients: seventeen years of experience and 1200 outpatient visits.Circulation.20011032125852590[CrossRef][PubMed]1138272810.1161/01.cir.103.21.25853.MurayamaHMaedaMSakuraiHUsuiAUedaYPredictors affecting durability of epicardial pacemaker leads in pediatric patients.J Thorac Cardiovasc Surg.20081352361366[CrossRef][PubMed]1824226910.1016/j.jtcvs.2007.09.0024.FortescueEBBerulCICecchinFWalshEPTriedmanJKAlexanderMEPatient, procedural, and hardware factors associated with pacemaker lead failures in pediatrics and congenital heart disease.Heart Rhythm.200412150159[CrossRef][PubMed]1585114610.1016/j.hrthm.2004.02.0205.Beaufort-KrolGCMulderHNaqelkerkeDWaterbolkTWBink-BoelkensMTComparison of longevity, pacing, and sensing characteristics of steroid-eluting epicardial versus conventional endocardial pacing leads in children.J Thorac Cardiovasc Surg.19991173523528[CrossRef][PubMed]1004765610.1016/s0022-5223(99)70332-66.OdimJSuckowBSaediBLaksHShannonKEquivalent performance of epicardial versus endocardial permanent pacing in children: a single institution and manufacturer experience.Ann Thorac Surg.200885414121416[CrossRef][PubMed]1835553710.1016/j.athoracsur.2007.12.075

## Figures and Tables

**Table 1:** Comparison of Automatic Atrial Threshold Algorithms Across Major Device Manufacturers

Manufacturer	Automatic Atrial Threshold Algorithm	Nominal Programming	Ventricular Lead Function During Atrial Threshold Testing	RV Lead Mandatory for LV Pacing
Abbott Laboratories (Chicago, IL, USA)	ACap Confirm™	Monitor	Biventricular	Yes
Biotronik (Berlin, Germany)	Atrial Capture Control™	On	RV-only on most models*	Yes
Boston Scientific (Natick, MA, USA)	PaceSafe Right Atrial Automatic Threshold™	Monitor	RV-only	Yes
Medtronic (Minneapolis, MN, USA)	Atrial Capture Management™	On	RV-only on most models*	Yes

RV: right ventricular; LV: left ventricular.

*Biventricular pacing available on the Enitra™ (Biotronik, Berlin, Germany) and the Percepta™, Serena™, and Solara™ (Medtronic, Minneapolis, MN, USA) devices.
